# Revenge among Parents Who Have Broken up Their Relationship through Family Law Courts: Its Dimensions and Measurement Proposal

**DOI:** 10.3390/ijerph16244950

**Published:** 2019-12-06

**Authors:** Miguel Clemente, Dolores Padilla-Racero, Pablo Espinosa

**Affiliations:** Department of Psychology, Universidade da Coruña; 15071 A Coruña, Spain; miguel.clemente@udc.es (M.C.); dolores.padilla@udc.es (D.P.-R.)

**Keywords:** moral disengagement, measurement, parent–child relationships, dark triad, revenge

## Abstract

This work analyzes inter-parental revenge after a breakup process, as it relates to the dark triad of personality, moral disengagement, and sex role ideology. Our objective was to test a predictive model for revenge based on these variables. Additionally, a scale to measure revenge among parents was developed. A sample of 384 participants who had minor children, had broken up their relationship, and had undergone or were undergoing problematic judicial procedures regarding their children completed a survey. They answered to measures of the dark triad of personality (Machiavellianism, narcissism, psychopathy); moral disengagement strategies; sex role ideology, and revenge. An instrument (the R scale), with adequate reliability and validity was developed to test revenge. Results show that revenge behaviors are perpetrated by 1 to 5% of participants. Revenge has three components: revenge through the child, revenge through economic manipulation, and revenge by cutting off communication. Results also show that for males, but not for females, sex role ideology mediates the relationship between the components of the dark triad, moral disengagement, and revenge. This paper aims at providing some insight for the protection of minors from manipulation by means of the Family Courts.

## 1. Introduction

Revenge is one of the main causes of the perpetration of crimes. It even becomes the root cause of the crimes of stalking and sexting, for example, but it also explains most homicides [1]. It is a global phenomenon, and it manifests in practically all societies and it endures over time, so that it is sometimes practiced even though years have elapsed since the occurrence of the event that was considered injurious by a person or group. Revenge manifests in very different ways according to whether it is carried out by a single individual or a group [2]. It is more intense when at the individual level. In group revenge, there must be entitativity, which serves to unite the group.

One of the possible definitions of revenge is provided by Jackson, Choi, and Gelfand [3], who consider it as an aggression, and therefore a behavioral response emitted by an individual or a group. It is based on the perception of an injustice suffered by the individual or group—which becomes the aggressor—and it is directed against the person that the aggressor identifies as the cause of the injury suffered. Therefore, although revenge is behavioral, it is essential for the person seeking revenge to have a perception of having been hurt, and to perceive that the harm they believe they have suffered is not involuntary [4,5]. This may be why revenge and anger are usually closely related [6], and why anger is a good predictor of revenge [7]. The relationship between anger and revenge has resulted in research relating the three variables that currently make up the so-called dark personality triad (dark triad) to revenge. This research found evidence of the relationship between narcissism and revenge [8,9,10].

Revenge has been conceived as an alternative mechanism to the justice system. Some authors state that the justice system has not "subjectively" restated the alleged victim [3]. However, in cases of conflict between parents (Family Law,) this resource is used even if the Justice System also intervenes, as is specified below [11,12]. Perhaps one of the characteristics of revenge is that it may be carried out despite the Justice System’s actions and this may be due to the fact that the future aggressor considers that the harm done must be directly repaired, regardless of the actions of the law and society. This leads to the conception of revenge as something that must be harmful, aimed at inflicting pain on others, and it is accompanied by rancor, a concept that is excluded from penal codes, but not from personal ones.

The idea of revenge, and especially when directed to harm, is opposed to that of forgiveness, a concept that is more religious than psychological, and which has been addressed particularly by positive psychology [13,14]. The concept of forgiveness has not been well-defined from a psychological point of view, even though Casullo [15] had already addressed it and created a scale (CAPER), retaking the concept used previously by Kaminer, Stein, Mbanga, and Zungu-Dirwayi [16], and labeling it as a process in which the motivation to retaliate decreases, along with the negative emotions felt toward the person considered to be the aggressor. These authors also state that negative emotions turn into positive emotions, such as compassion or benevolence. Thus, Adam-Karduz and Saricam [13] showed that positivity, forgiveness, happiness are all positively related to one another, and the three correlate negatively with revenge. The same argument was defended by Garzón-Arañón and Barahona-Esteban [14].

The functionality of revenge and what the retaliator “gains” has come under much debate [17,18]. However, the literature indicates that there is no evidence that revenge generates any personal benefit for the avenger [3,19,20]. As far as Family Courts are concerned, some authors conceive the use of the Justice System itself as a way of attacking the other parent [21], such that an individual aggression takes place using the very system, which becomes an accomplice of the aggression. Such aggression usually occurs in people who obtain high scores in the components of the dark triad, but the main predictor is a high score in the variables that imply moral disengagement [22]. Both moral disengagement and the components of the dark triad are part of what Moshagen, Hillbig, and Zettler [23] called the "dark personality factor," which is the general tendency to disregard other people’s well-being in favor of self-interest, independently of the fact each one of the traits that make up the dark factor may have other specific effects. These people’s reasons for revenge usually originate in the fact that the other partner was the one who cut off the couple relationship [12], and indirect revenge is sought, using the children they have in common. This perspective is interesting from the viewpoint of forensic psychology, as parents who score high on the Judicial Manipulation Scale created by Clemente, Padilla-Racero et al. [21] are prone to manipulate their children, occasionally committing physical and sexual aggression and sometimes even provoking the child’s death, as a way of seeking revenge on the other parent.

In fact, revenge tends to be more intense if the supposedly injured party is close to the person perceived as the aggressor, especially if this person was his or her romantic partner [24,25]. Moreover, when revenge is carried out by people who were emotionally close, in addition to judicial harassment [21], they use all kinds of elements within reach, such as gossip, hurtful remarks to acquaintances, coercive actions such as persecutions, etc. [26,27]. The aggressors seize the concept of "virtuous violence" [28], as a form of moral disengagement, such that they perceive that the only way left for them is revenge. Furthermore, they seek and obtain the support of people from their environment, so that revenge becomes a social and moral obligation, even protected by social values. On the other hand, it has been argued that the Justice Systems of societies in which there is a greater culture of honor tend to tolerate the male practices of attacking women [18,29,30,31]. This process of revenge has a limit, which commonly takes place when the retaliator’s target is more powerful than the avenger, although frequently the opposite is what happens; that is, the avenger (besides believing he or she has suffered harm) is usually the one with more power [29,32].

This research focuses on one parent’s revenge on the other parent after a divorce process, in an attempt to determine the extent to which such violence is considered appropriate by potential avengers, the possible components of such revenge, and whether sex role ideology mediates between the dark triad and revenge.

It is hypothesized that one of the main components of revenge will include the utilization of the common children (Hypothesis 1). Another hypothesis is that the cognitive indicators related to the dark personality factor (moral disengagement, narcissism, psychopathy, and Machiavellianism) will predict favorable attitudes toward revenge (Hypothesis 2). Finally, it is hypothesized that sex role ideology plays a different role in revenge for men and women. Through a mediation model, we can explore the relationship between the variables of the dark factor and revenge and determine whether this relationship is mediated differentially by sex role ideology in men and women (Hypothesis 3). Additionally, we predict that revenge behaviors allow the possibility of creating a scale, the "R" scale. The psychometric properties of this scale are verified.

## 2. Materials and Methods

### 2.1. Participants

Participants in this study were 384 individuals, all of them residing in the region of Galicia, in northwestern Spain. Concerning sex, 31.8% of the sample were male and 68.2% were female. The average age was 43.4 years (*SD* = 6.96), ranging from 27 to 62 years. With regard to the level of studies, 1.6% of people had no studies, 11.7% had primary or secondary studies (practically all had secondary studies), 44.5% had high school studies, and 42.2% had university studies. It was previously verified that all participants had children and, that at the time of completing the scale, one of the children was a minor, that they had broken up with the other parent (with whom they had at least one child), and that they had been involved in judicial family processes because they had not reached an agreement about the type of custody or the visitation schedule. Concerning the children, 57% of the participants had one child, 35.3% had two, and 4.7% had three. All these data indicate that the sample’s social and demographic characteristics are very similar to those of the Spanish population.

The sample was non-random and incidental. The surveyors were university students of several subjects, who worked altruistically. To verify the accuracy of the information collected by the surveyors, respondents provided a telephone number, and they were contacted and asked about some of their responses. All participants previously signed an informed consent, in which we specified the purpose of the investigation and requested their participation and permission to publish their data, preserving their anonymity. They were guaranteed anonymity, and the data were processed so that the respondent could not be identified.

### 2.2. Material

In addition to the above questions to determine that participants met the selection prerequisites (minor children, couple breakup, and the existence of legal problems because of the children), the following tests were applied to determine their sociodemographic characteristics.

The MAC-IV Machiavellianism Scale [33]. This version has 20 items rated on a 7-point scale. Nine items belong to the subgroup of Manipulation Tactics, 9 are included in the group of People’s Views, and 2 items are part of the group of Moral Principles. Although the scale is divided into three subgroups, when scoring, all 20 items of the scale are added. Its psychometric properties for Spanish populations have been widely evaluated [34,35,36,37]. Sample items are: "The best way to deal with people is to tell them what they want to hear" or “Most people are basically good and kind” (reverse scored. Its mean reliability score in the original study was 0.79.

The Narcissistic Personality Inventory (NPI) by Raskin and Hall [38]. This scale comprises 40 items rated on a 6-point Likert type response format. It measures the following facets of narcissism: Authority (8 items), Exhibitionism (7 items), Superiority (5 items), Entitlement (6 items), Exploitation (5 items), Self-sufficiency (6 items), and Vanity (3 items). Sample items are “I have the skill to influence others” or “I will be a success.” The reliability score provided by the authors is 0.72. We used the Spanish version of the scale, whose psychometric properties were verified by García-Garduño and Cortés-Sotres [39].

Levenson’s Primary and Secondary Psychopathy Scales (LPSP) of Levenson, Kiehl, and Fitzpatrick [40]. This scale is composed of 26 items. The first 16 items measure Primary Psychopathy, and the last 10 measure Secondary Psychopathy. The scale is rated on a 5-point Likert type format. In the original study, the alpha coefficient for primary psychopathy was 0.82 and for secondary psychopathy 0.63. A meta-analysis of psychopathy scales, including this one, can be found in Salvador, Arce, Rodriguez-Diaz, and Seijo [41]. Sample items are: "Success is based on the survival of the fittest; I’m not worried about losers" (primary psychopathy) and “I don’t plan anything very far in advance” (secondary psychopathy).

The of Sex role Ideology Scale of Moya, Navas, and Gómez [42] is based on the works of Glick and Fiske [43] on ambivalent sexism, which indicates two components of sexism: traditional sexism, called hostile sexism, which refers to a negative attitude toward women; and benevolent sexism, which implies the stereotyped consideration of women that constrains them to a series of roles, but that has a positive affective tone toward them, and also attributes prosocial characteristics to them. A reduced 12-item version was used. It explores people’s beliefs about the roles and behaviors that men and women should perform and about the relations that the sexes should have with each other [44]. Its reliability ranges from 0.71 to 0.82. Sample items are: "Although some women like to work outside home, it should be the man’s responsibility to provide economic support for the family” and “It is natural that men and women perform different tasks”.

Scale of moral disengagement. To measure moral disengagement, we used the Propensity to Morally Disengage (PMD) scale of Moore, Detert, Treviño, Baker, and Mayer [45] which was designed for adults in any type of context. It comprises 24 items rated on a 7-point Likert scale ranging from strongly disagree to strongly agree. The items measure each of the eight moral disengagement strategies proposed by Bandura Barbaranelli, Caprara, and Pastorelli [46]. Examples of items I the scale are: "It is alright to lie to keep your friends out of trouble" or “In contexts where everyone cheats, there’s no reason not to.” In the original study [45] authors report a reliability of 0.90. Furthermore, it has a low correlation with social desirability measures, so it is not prone to be contaminated by this bias.

We elaborated an ad hoc test for this research, which is included as an annex. Subjects are asked to think about what is happening to them before reading each item, and they are requested to respond whether or not they would agree to carry out the actions described. Fifty actions were selected, all related to disputes between parents and ways of taking revenge on the other member of the couple, some of them using the children, and some even related to possible sexual aggression with the aim of harming the other parent. The statements were created by a group of four experts, two psychologists and two social workers, who were employees of the Spanish Family Courts, and whose role was to advise the judges about the emergence of family problems. All statements had to be unanimously approved by the four experts, who selected 38 statements. The response format was a 5-point Likert type scale, ranging from totally disagree to totally agree.

### 2.3. Procedure

The surveyors located the potential participants, requesting their cooperation in two shopping centers in the Spanish region of Galicia. Surveys were collected on eight weekends. The people who agreed to participate were asked to sign an informed consent and were then requested to complete the questionnaires. They were thanked for their collaboration after they finished filling in the psychological tests.

This research was approved by the Ethics Committee of the corresponding author’s University (Project Identification code 04/19). It complies with the Helsinki criteria and with the ethical principles of the American Psychological Association (APA).

### 2.4. Statistical Analysis

First, we calculated the reliability scores for the scales in the study. We also checked for gender differences using *t*-tests. As for the questionnaire developed for this study, we examined the frequency of agreement with the actions described and carried out exploratory (EFA) and confirmatory (CFA) factor analyses to test its internal structure. Additionally, we checked the resulting factors’ and the scale’s reliability. Finally, a SEM model was estimated to verify the effect of the variables that make up the dark personality factor on revenge, using as a mediator sex role ideology. The participants were divided by sex to examine the possible effects of sex on the relationship between variables. To obtain an indicator of the dark factor, we created a variable in the SEM from all the relevant variables used in the study: Machiavellianism, Psychopathy, Narcissism, and Moral Disengagement. Although the dark factor has been described not only based on these variables, it is a robust construct and its predictive capacity is maintained even if important indicators are eliminated [23]. As chi-square is influenced by the sample size [47] and the size of the model [48], for CFA, we used other statistics to measure the goodness-of-fit, such as the comparative fit index (CFI), the standardized root mean square residual (SRMR), and the root mean square error of approximation (RMSEA). Zero-order correlations were calculated for all the variables in the model, which also served as an indicator of the concurrent validity of the revenge scale.

## 3. Results

### 3.1. Reliability of the Scales and Gender Differences

Overall, the reliability scores for the scales in the current study were acceptable. The lowest alpha score was for the MAC-IV scale (α = 0.64). LPSP global scale showed an alpha score of 0.82, while the alpha for the Primary Psychopathy scale was 0.78 and for Secondary Psychopathy 0.65. The NPI scale showed an alpha of 0.79, Sex Role ideology scale had an alpha of 0.86, and the PMD questionnaire showed the highest reliability (α = 0.93). These reliabilities are comparable to those reported by the authors.

We also checked for gender differences in the scales. No gender differences were found for Narcissism, Machiavellianism, sex role ideology, or secondary psychopathy. The only significant differences were found for moral disengagement (t(382) = 3.89, p < 0.001; males: M = 2.62, SD = 1.14; females: M = 2.21, SD = 0.89) and for primary psychopathy (t(382) = 4.28, p < 0.001; males: M = 2.64, SD = 0.77; females: M = 2.31, SD = 0.66).

### 3.2. Acceptance of Revenge Procedures

The subjects’ responses showed that very few subjects would agree to perform the revenge actions that were proposed in the questionnaire, with most participants marking the cell that stated that they would never do that. Results indicate that the behavior that obtained a highest degree of agreement ("Asking for the guardianship and custody of the child just to stop paying alimony to your ex-partner") was accepted by 5.2% of the respondents, whereas the least accepted behaviors (e.g., “Sexually assaulting your daughter, thrusting objects into her anus or vagina, so that she will bleed, but, in the case of the vagina, not too deep so that her hymen will not tear, and then say that she does it herself") would only be carried out by 0.8% of the respondents. This shows that the utilization of children is not accepted by a majority, but it is worrying that 5% of the parents are willing to use their children to take revenge on the other parent, and that there is even about 1% of parents who are willing to sexually abuse them, causing serious damage.

Table S1 shows the degree of acceptance of revenge by item No significant differences were found between men and women in any of the variables of the Revenge Scale.

### 3.3. Components of Revenge

Factor analysis was performed to determine whether revenge is a single variable or has several components. First, EFA was performed (principal component and varimax rotation), which yielded three factors. The Kaiser-Meyer-Olkin sampling adequacy measurement obtained a very high value (0.959) and Bartlett’s sphericity test was significant, χ^2^(703) = 22993.00, *p* < 0.001. The model explained a global variance of 73.75% and the three factors obtained are detailed below:

Factor I explained 45.27% of the variance. The items that mainly load on this factor refer to forms of revenge that even include the perpetration of sexual abuse, as well as convincing people from their environment, especially relatives on their side of the family, to attack the other parent. It has been labeled "Revenge through children and third parties.” It comprises 27 items.

Factor II explained 15.05% of the variance and contains six items. The items related to this factor refer to revenge through economic manipulation. It includes all attempts to avoid providing the economic pension that corresponds to the child, as well as to convince the child of the injustice of having to pay that money. It implies the use of an economic harassment toward the other parent. It has been called "Revenge through economic manipulation.”

Factor III explained 13.43% of the variance. It has five items and refers to extreme behaviors that involve cutting off any type of negotiation with the former partner, and also includes threats and cutting off communication with common acquaintances or using all the resources and time needed to take revenge on the ex-partner. It has been called the factor as "Revenge by cutting off communication.” Table S2 shows the components of revenge in the "R” scale

These factors were verified through CFA, replicating the structure of the EFA. The CFA for the three-factor model obtained acceptable goodness-of-fit indices (RMSEA = 0.092, SRMR = 0.044, CFI = 0.922). Every item showed significant regression weights for the proposed factors. In addition, a CFA was carried out for the main factor obtained in the EFA (Revenge through children and third parties). In this case, the goodness-of-fit indices were very satisfactory (RMSEA = 0.047, SRMR = 0.017, CFI = 0.991). 

### 3.4. Internal Reliability of the “R” Scale

Although the proposed “R” scale of revenge explains an adequate amount of variance, we wished to determine the correlations between the items of the global scale and the corresponding subscales. For this purpose, the Cronbach alpha index, as well as Friedman’s ꭓ^2^ and its level of significance, were calculated. As can be seen in Table 1, the values of chi-square were significant. As Table 1 shows, alpha values were acceptable for the scales corresponding to Factors II and III, and very high for Factor I and the total scale.

### 3.5. Dark Factor, Sex Role Ideology, and Revenge

**Figure 1:**
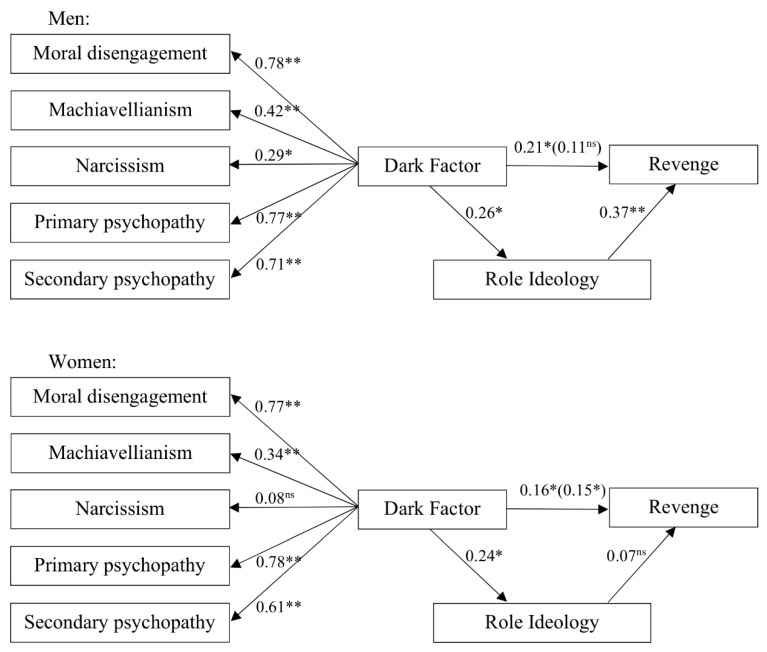
Mediation model for the Dark Factor, sex role ideology and revenge. * *p* < 0.05. ** *p* < 0.01; ^ns^ = non-significant; χ^2^(18) =25.28, *p* = 0.12, root mean square error of approximation (RMSEA) = 0.033, standardized root mean square residual (SRMR) = 0.045, comparative fit index (CFI) = 0.98. Direct effects shown. Total effects in parentheses.

The SEM indicated that sex role ideology mediated the relationship between the dark factor and revenge in the case of men, but not in the case of women. The model displayed in Figure 1 reveals these sex differences when conceiving of revenge.

There was a significant total effect of the dark factor on revenge both for men and women. However, in the case of men, we found that the direct effect was not significant (discounting the indirect effects explained by sex role ideology). In the case of women, this effect was not produced, and sex role ideology was not a significant predictor of revenge. In fact, the critical ratios for the differences between the parameters indicate that the relationship between sex role ideology and revenge was significantly different in men and women (*z* = 3.23, *p* < 0.01). There were no significant sex differences in the rest of the parameters of the model. Table 2 shows the correlations between the different variables of the model.

## 4. Discussion

The results of this work provide support for the objectives of the study. Revenge against the other parent after a couple break-up is manifest to a low, albeit statistically (and socially) important extent. Although most parents reject revenge, in some cases, around 5% of vengeful people attack those who they think have attacked them. That is, 1 out of every 20 people would not have any qualms about taking revenge on their ex-partner, (although in the case of those actions that involve sexual aggression of the children, that percentage is much lower, somewhat less than 1%).

To a large extent, revenge behaviors involve minor children. In fact, the factor that explains a higher percentage of variance and has a better fit within the CFA is related to the use of the children, which provides support for our first hypothesis. Revenge does not appear to be an unitary concept, at least not as regards family procedures. In these cases, it has three components: revenge through the utilization of the child and third parties, revenge through economic manipulation, and revenge by cutting off communication. Accordingly, we propose the "R" scale as a measure of revenge in family procedures. It shows adequate reliability (very high alpha values) and concurrent validity (correlates with dark factor traits).

We also found that revenge is related to the variables of the dark triad and moral disengagement, through the dark personality factor as expected in the line of our second hypothesis. Finally, it was hypothesized (Hypothesis 3) that a mediation model could be created in which the relations between the variables of the dark triad, moral disengagement, and revenge would be differentially mediated by sex role ideology as a function of sex, and the results support this hypothesis. The proposed SEM model shows that the dark factor, which represents the common tendency to act to the detriment of others to achieve personal goals, is a significant predictor of revenge. Except for narcissism, each of the components used in this study of the dark factor correlate significantly with revenge. However Egan, Hughes, and Palmer [49] already argued that narcissism is the "lighter" factor within the dark triad, and that its correlation with other indicators of negative behavior is not as high as that of Machiavellianism or Psychopathy, so it is not surprising that, in our study, it was not a significant predictor.

On another hand, although there are no differences between men and women in the magnitude or type of vengeful behaviors, the explanation for revenge in each sex differs. For men, sex role ideology explains the relationship between the dark factor and revenge, because it mediates the relationship between the two variables, which does not occur in the case of women. Possibly, men interpret their attitudes of revenge through the sex role ideology, whereas women use other cognitive mechanisms. There is no doubt that revenge is based on behavioral tendencies related to the dark factor, but in the case of men, sex role ideology seems to serve as a vehicle to explain the tendencies of revenge determined by the dark factor as a common core of anti-social attitudes to the detriment of others. These results are not surprising, as sex role ideology includes attitudes of antagonism toward women, which, in this case, seem to channel the negative tendencies of men’s behavior. It would be of interest to determine which cognitive variables mediate in the relationship between this common core and revenge in the case of women.

The results allow us to take another step in conceiving of some parents’ harassment strategies toward their ex-partners, and place them within a concept clearly linked to the dark triad. This idea is related to the works of Jackson, Choi, and Gelfand [3], who specify that revenge is an alternative mechanism to the Justice System, and this leads to another step, confirming that some people are capable of using the Justice System to pervert its function of "justice" and turn it into a weapon to attack those whom they want to injure. This notion led Clemente et al. [21] to create a scale to measure judicial harassment. Some previous research in Psychology and Family Law has proposed that, sometimes, one parent harms the children psychologically or physically in order to attack the other parent. When the other parent observes this maneuver, he or she is obliged to denounce the ex-partner and forced to prove an aggression that is usually impossible to verify because it is carried out in private. Thereby, the complaint turns against the plaintiff and he or she becomes the defendant [11,12]. The percentage of parents who are capable of using revenge procedures against the other parent had not been determined in previous research.

Undoubtedly, using one’s own children to take revenge on the other parent, even perpetrating sexual aggression, is clearly related to the dark triad personality, as well as to the mechanisms of moral disengagement, in turn, all components of the dark personality factor [22]. For this purpose, perpetrators use the process of objectification, dehumanizing the ex-partner and the children, and the need to avenge themselves for being humiliated comes before everything else. Padilla-Racero and Clemente [12] established that such a thought tends to originate in the fact that the person who is supposed to have offended did so when breaking up the relationship. If it was the woman who broke off, she attacked the man, who considers that he can leave his partner, but that this option is denied to women. This phenomenon is often referred to as "virtuous violence" [28], a concept that coincides with the sad fact that the more patriarchal a society is, the more the women are attacked within a couple relationship, but most especially after the break-up of the relationship, as this break-up is conceived by the man as a grievance.

Another important issue is that, of all the areas in which revenge may occur, one of the special relevance is that of the couple’s break-up because, as Chester and De Wall [24] established, revenge is more intense if the person who feels hurt is close the person perceived as the aggressor, and especially if this person is his or her romantic partner [18,29,30,31]. Unfortunately, the data on the deaths of mothers and children at the hands of the male parent provide support to this explanation.

Harassment and revenge often go hand-in-hand, and the fact of the three components of revenge found in this work proves this. Avengers use their children, manipulate people in the environment, and carry out economic harassment. Economic harassment is a clear example of how the justice system is used, as the parent with the greatest economic resources can pay long and costly judicial proceedings, whereas the low-income parent has great difficulty to defend him- or herself [21].

Results show that parents capable of lying in court about their partner to the extent of manipulating their children score higher in the dark factor of personality. In particular, they are more morally disengaged and show higher sub-clinical primary psychopathy, since both are the highest loading variables for the dark factor. These variables, together with sex role ideology in the case of men, are good predictors of revenge, as measured by the scale proposed in this study. Thus, the current study provides evidence that these “dark personality” variables are actually related to revenge in a family court setting. As a practical implication, our results may help outline a profile of which dark traits are more closely related to revenge. This evidence may help professionals make decisions based on “dark traits” of litigating parents as they constitute a risk for revenge and illegitimate manipulation of the judicial process. Conversely, other variables like Narcissism contribute less to the core of the dark factor, and are subsequently less related to revenge. It is of paramount importance to scientifically determine who is more prone to be a judicial manipulator and the present revenge scale constitutes a good criterion to test variables that increase this risk.

Some limitations of this study ought to be addressed in future research. Bigger and random samples would help refine the scale. It would also be interesting to test it with parents litigating during data collection, instead of parents recollecting their past experiences, and to be able to contrast revenge scores to actual behavior and third parties reports. However, our current results may contribute to detect parents who pose a risk for their siblings and to better protect the children’s welfare.

## 5. Conclusions

This research presents the advantage of using a broad sample of people who have faced judicial procedures in Family Law and provides not only an advance in determining how much and by whom is revenge used, but also the components of revenge. It is especially useful professionally as it provides a measurement instrument, the so-called "R" scale, whose psychometric properties have been verified. However, it also presents some limitations. The measurement scales used, especially the Mach-IV, have been criticized. The set of tests applied required a long time for completion, and the proposed scale could have been confirmed using more rigorous statistical procedures, although it would have been necessary to have larger samples. The fact that the sample is incidental is another important limitation. Nevertheless, we believe that this work provides a significant theoretical and professional advancement.

## Figures and Tables

**Table 1:** Reliability of the scale and its factors.

	Friedman’s χ^2^ (*df*) *	*p*	*α*
Total scale	770.32 (383.37)	0.001	0.98
Factor I	159.67 (383.26)	0.001	0.99
Factor II	46.00 (383.5)	0.001	0.84
Factor III	31.57 (383.4)	0.001	0.85

* Chi-square (degrees of freedom).

**Table 2:** Correlations among the variables included in the model.

	Revenge	Role Ideology	Machiavellianism	Primary Psychopathy	Secondary Psychopathy	Narcissism
Role Ideology	0.20 **					
Machiavellianism	0.08	0.22 **				
Primary psychopathy	0.14 *	0.15 **	0.28 **			
Secondary psychopathy	0.09	0.07	0.30 **	0.51 **		
Narcissism	0.03	0.06	0.17 **	0.38 **	0.10 *	
Moral disengagement	0.15 **	0.24 **	0.27 **	0.62 **	0.49 **	0.25 **

*N* = 384. *p < 0.05. **p < 0.01.

## Supplementary Materials

The following are available online at https://www.mdpi.com/1660-4601/16/24/4950/s1. Table S1: Degree of acceptance of revenge by item. Table S2: Components of revenge and the "R” scale.
