# Supplementary material for: Revenge among Parents Who Have Broken up Their Relationship through Family Law Courts: Its Dimensions and Measurement Proposal
Source: Int J Environ Res Public Health. 2019 Dec 6;16(24):4950. doi: 10.3390/ijerph16244950 (PMC6950745; doi:10.3390/ijerph16244950)
Supplement: Supplementary file 1 [file ijerph-16-04950-s001.pdf]

Supplementary material. Tables.

Table S1

Degree of acceptance of revenge by item

	% Agreement	*M*	*SD*
4. Asking for the guardianship and custody of the child just to stop paying alimony to your ex-partner	5.2	1.47	0.92
5. Explaining to your child that the situation you are undergoing is very bad for you, and that it is the ex-partner's fault, who also decided to break up the relationship and, therefore, has provoked everything	3.6	1.36	0.76
17. If your child does not want to visit you, you ask the court to order the police force to take the child away from your ex-partner when you have the right to see the child and hand the child over to you for the visit.	2.6	1.32	0.71
35. Try to keep common acquaintances from contacting your ex-partner	2.6	1.27	0.69
2. Convince your child that alimony is just for him/her to spend as he/she wishes, and that your ex-partner is misappropriating it.	2.3	1.35	0.72
7. Speaking ill of your ex-partner to everyone, so that everyone knows that his/her decision has ruined your life	2.1	1.30	0.68
3. Refusing to pay, beyond alimony, any health or education expenses for your child	2	1.29	0.69
21. Convincing your child that he/she has another name to be used at your ex-partner's home, to annoy your ex-partner	2	1.13	0.44
9. Spreading false rumors about your ex-partner that harm him/her	1.8	1.17	0.56
22. Threatening to separate your ex-partner from your child	1.8	1.23	0.63
31. As you know that your ex-partner is annoyed because your child does not study or do his/her homework, you return the child to your ex-partner after the weekend without having studied or done his/her homework	1.8	1.11	0.41
16. Requesting the courts for the guardianship and custody of your child; that is, for the child to live with you and your parents, although this does not really matter to you, but you know that to separate your child from your ex-partner is what would hurt him/her the most	1.6	1.21	0.58

19. Continually denouncing in Court that your child rejects you because of your ex-partner, who manipulates the child, and asking the Court to punish your ex-partner and your child for this.	1.6	1.17	0.54
37. Using all your resources to take revenge on your ex-partner.	1.6	1.17	0.54
8. Talking to your ex-partner's parents and trying to win them over and make them think that their son or daughter is a bad person	1.5	1.20	0.58
36. Waiting as long as it takes, even years, to take revenge on your ex-partner.	1.5	1.18	0.59
1. Stop sending alimony for your child to your ex-partner, even though you are obliged to by a judge, and with the risk of being fined or even arrested.	1.3	1.22	0.55
6. Taking advantage of any moment to speak ill to your child about your ex-partner	1.3	1.20	0.53
14. Calling up your ex-partner by phone with a hidden number late at night	1.3	1.15	0.49
15. Whenever you can, denouncing in Court that your ex-partner treats your child badly, does not feed the child properly, does not care for the child properly, etc., even if you know it is a lie.	1.3	1.13	0.51
23. Threatening your ex-partner with revenge, using your child	1.3	1.14	0.49
18. Inventing and informing the Court that, when you pick up the child, he/she has signs of having been beaten, to accuse your ex-partner and annoy him/her.	1.1	1.12	0.46
20. Requesting the Court to send your child to a juvenile center to remove the child from your ex-partner.	1.1	1.11	0.44
25. Destroying your child's clothes when you have him/her for the weekend, so that your ex-partner will be afraid of what you might do to the child, and then saying it is your own child who destroys the clothing, and you can do nothing [to prevent this].	1.1	1.10	0.42
26. Hitting your child lightly, so he/she will have bruises and signs of blows, and then saying that the child is very restless and is always falling down, so that it cannot be proven that it was you.	1.1	1.11	0.45
29. As you know that your ex-partner gets annoyed when the child is not clean, after the weekend, you return the child very dirty, without having bathed on any day.	1.1	1.12	0.45
30. As you know that your ex-partner gets annoyed when the child does not eat well, you feed him/her only sweets.	1.1	1.12	0.45

11. Contacting your ex-partner's neighbors to tell them your version and criticize your ex-partner.	1	1.15	0.49
13. Painting graffiti with insults to your ex-partner on the walls of the house where your ex-partner and your child live.	1	1.12	0.47
28. In the event that you have assaulted your daughter, saying she does it herself, manipulated by your ex-partner, because what your ex-partner wants is to cut off the visits with your daughter, and she provokes the child to self-injure herself.	1	1.12	0.44
10. Going to your child's school and speaking ill of your ex-partner to the teachers and all the staff of the center	0.8	1.14	0.45
12. Whenever possible, damaging your ex-partner's things, for example, scratching his/her car, puncturing the wheels, or soiling the clothes that are hung out, to which you have access when he/she opens the door for you to pick up the child	0.8	1.13	0.45
24. Harassing your ex-partner whenever you can, following him/her with your car so he/she will feel threatened.	0.8	1.12	0.44
27. Sexually assaulting your daughter, thrusting objects into her anus or vagina, to make her bleed, but in the case of the vagina, not too deep, so as not to tear the hymen, and then saying that she (your daughter) does that herself.	0.8	1.09	0.39
32. If your child gets sick or suffers an accident, you do not take her to a medical service to annoy your ex-partner.	0.8	1.10	0.44
33. Asking your current partner to help you to try to annoy your ex-partner.	0.8	1.14	0.48
34. Asking other people to lie to harm your ex-partner.	0.8	1.14	0.44
38. Not hesitating to use your child whenever necessary to annoy your ex-partner	0.8	1.11	0.45

Table S2

Components of revenge and the "R" scale

	FACTOR	F. I: 45.27%	F. II: 15.05%	F. III: 13.43 %
1. Stop sending food alimony for your child to your ex-partner, even though you are obliged to by a judge, and with the risk of being fined or even arrested.	II	.29	.65	.04
2. Convincing your child that alimony is just for him/her to spend as he/she wishes, and that your ex-partner is misappropriating it.	II	.13	.71	.07
3. Refusing to pay, beyond alimony, any health or education expenses for your child	II	.25	.73	.10
4. Asking for the guardianship and custody of the child just to stop paying alimony to your ex-partner	II	.02	.75	.24
5. Explaining to your child that the situation you are undergoing is very bad for you, and that it is the ex-partner's fault, who also decided to break up the relationship and, therefore, has provoked everything	II	.26	.70	.24
6. Taking advantage of any moment to speak ill to your child about your ex-partner	I	.58	.49	.25
7. Speaking ill of your ex-partner to everyone, so that everyone knows that his/her decision has ruined your life	II	.36	.63	.29
8. Talking to your ex-partner's parents and trying to win them over and make them think that their son or daughter is a bad person	I	.60	.51	.14
9. Spreading false rumors about your ex-partner that harm him/her	I	.65	.42	.29
10. Going to your child's school and speaking ill of your ex-partner to the teachers and all the staff of the center	I	.79	.38	.20
11. Contacting your ex-partner's neighbors to tell them your version and criticize your ex-partner.	I	.82	.35	.20
12. Whenever possible, damaging your ex-partner's things, for example, scratching his/her car, puncturing the wheels, or soiling the clothes that are hung out, to which you have access when he/she opens the door for you to pick up the child	I	.68	.30	.32
13. Painting graffiti with insults to your ex-partner on the walls of the house of your ex-partner and your child.	I	.89	.29	.22
14. Calling up your ex-partner by phone with a hidden number late at night	I	.79	.28	.29

15. Whenever you can, denouncing in Court that your ex-partner treats your child badly, does not feed the child properly, does not care for the child properly, etc., even if you know it is a lie.	I	.78	.26	.27
16. Requesting the courts for the guardianship and custody of your child; that is, for the child to live with you and your parents, although this does not really matter to you, but you know that to separate your child from your ex-partner is what would hurt him/her the most	I	.58	.39	.36
17. If your child does not want to visit you, you ask the court to order the police force to take the child away from your ex-partner when you have the right to see the child and hand the child over to you for the visit.	III	.43	.25	.45
18. Inventing and informing the Court that, when you pick up the child, he/she has signs of having been beaten, to accuse your ex-partner and annoy him/her.	I	.89	.23	.22
19. Continually denouncing in Court that your child rejects you because of your ex-partner, who manipulates the child, and asking the Court to punish your ex-partner and your child for this.	I	.67	.40	.29
20. Requesting the Court to send your child to a juvenile center to remove the child from your ex-partner.	I	.87	.23	.29
21. Convincing your child that he/she has another name to be used at your ex-partner's home, to annoy the ex-partner	I	.79	.27	.40
22. Threatening to separate your ex-partner from your child	III	.44	.27	.55
23. Threatening your ex-partner with revenge, using your child	I	.66	.21	.54
24. Harassing your ex-partner whenever you can, following him/her with your car so he/she will feel threatened.	I	.85	.25	.32
25. Destroying your child's clothes when you have him/her for the weekend, so that your ex-partner will be afraid of what you might do to the child, and then saying it is your own child who destroys the clothing, and you can do nothing [to prevent this].	I	.88	.24	.26
26. Hitting your child lightly, so he/she will have bruises and signs of blows, and then saying that the child is very restless and is always falling down, so that it cannot be proven that it was you.	I	.81	.20	.26
27. Sexually assaulting your daughter, thrusting objects into her anus or vagina, to make her bleed, but in the case of the vagina, not too deep, so as not to tear the hymen, and then saying that she (your daughter) does that herself.	I	.90	.22	.25
28. In the event that you have assaulted your daughter, saying she does it herself, manipulated by your ex-partner, because what your ex-partner wants is to cut off the visits with your daughter, and she provokes the child to self-injure herself.	I	.78	.21	.34
29. As you know that your ex-partner gets annoyed when the child is not clean, after the weekend, you return the child very dirty, without having bathed on any day.	I	.80	.20	.38

30. As you know that your ex-partner gets annoyed when the child does not eat well, you feed him/her only sweets.	I	.71	.24	.40
31. As you know that your ex-partner gets annoyed because your child does not study or do his/her homework, you return the child to your ex-partner after the weekend without having studied or done his/her homework	I	.79	.25	.37
32. If your child gets sick or suffers an accident, you do not take her to a medical service to annoy your ex-partner.	I	.83	.22	.30
33. Asking your current partner to help you to try to annoy your ex-partner.	I	.75	.28	.43
34. Asking other people to lie to harm your ex-partner.	I	.75	.25	.44
35. Trying to keep common acquaintances from contacting your ex-partner	III	.31	.42	.59
36. Waiting as long as it takes, even years, to take revenge on your ex-partner.	III	.43	.22	.73
37. Using all your resources to take revenge on your ex-partner.	III	.45	.14	.79
38. Not hesitating to use your child whenever necessary to annoy your ex-partner	I	.76	.22	.42
